# Sarcoma of the Tongue Treated with Pyoktanin

**Published:** 1894-10

**Authors:** E. S. Perman, L. G. Sellstedt


### SARCOMA OF THE TONGUE TREATED WITH PYOKTANIN.

By DR. E. S. PERMAN.

Translated from *Hygiea* by L. G. SELLSTEDT, Esq.

SARCOMA of the tongue is an uncommon disease, which only within the last ten years has begun to be recognized. Butlin, in his *Krankheiten der Zunge*, Vienna, 1887, says that he has only known of three cases of it. The first was one in New York, of a child three years old, published by Jacobi in 1870. The second was one treated by Goddle, in which the tumor was situated at the back of the tongue, and had reappeared after excision, and where afterwards metastasis of the skin had taken place. In the third case, Butlin had only seen pieces of the excised parts, and which he on investigation found to be round cells or sarcoma of the tongue. (A number of cases here follow in illustration of the subsequent history of the disease, which for want of space are omitted.) Continuing, Dr. Perman says, Dr. Hakanson has kindly given me the following history of the case :

Mrs. V. F., 30 years of age, called on him the first time, December 17, 1892, and told him then that, some time in May or June of the same year, she had once or twice observed streaks of blood in the saliva and also some unpleasant feeling in the throat. She applied to Dr. Gibson, her family physician, who touched it with a solution of nitrate of silver, as he could only find some reddening (in cav. orale). Her general health was good and there was no difficulty in swallowing.

During the Summer all went well, and it was only in the middle of December that she noticed something unpleasant in the throat. She again consulted Dr. Gibson, who, after examination with mirror, discovered a swelling in the root of the tongue and sent her to Dr. Hakanson. She was permitted to make an intended journey into the country over Christmas, and returned in the early part of January, 1893, when Dr. Hakanson found the tumor somewhat increased in size.

The 14th of January, 1893, in company with Dr. Hakanson, I made my first examination. When the tongue was drawn out, we could just see a tumor protrude at the root of the tongue. By the aid of a mirror the swollen mass was seen to be 1½ to 2 c. m. high and about as thick as the end of the little finger. The mucous membrane of its surface seemed to be raised and covered with mucus, not ulcerated. It hid the parts below, so that we could only perceive that the whole root of the tongue seemed to be elevated. It felt soft to the touch ; no induration in the surrounding tongue-tissue was felt.

The 17th of January, 1893, at the Sofia Home, a piece of the elevated tumor was removed with a galvano-caustic snare, by Dr. Hakanson, for microscopic examination. By the mirror examination it was seen that this only consisted of a part of a larger tumor, whose foundation involved the right half of the root of the tongue and over the median line, even reached a little way into the left half. It was uneven on the surface and seemed to be divided into smaller sections by incisions, of which the excised part constituted one of the most prominent. It seemed to be considerably elevated, 1½ to 2 c. m., over the level of the root of the tongue. The mucous membrane of it was not ulcerated. To the touch it felt as soft as its surroundings, nowhere fluctuating. Its lower margin seemed to extend almost as far as the epiglottis.

The general condition of the patient seemed to be good. In general she had no severe pain ; it only "felt unpleasant in the throat," besides which she was troubled with mucus in the throat. Latterly, however, she would often wake up at night, when the throat troubled her, and she had trouble to breathe. No difficulty in speaking nor in swallowing until lastly, when she was only able to take liquid food. There was no reason to suspect lues. Experimentally, large doses of iodide of potash were ordered, which she took for a week without result.

By examination on the 25th of January it was easy to perceive that the tumor had increased in size. The protruding mass of it seemed to be quite as big as a walnut and reached quite up against the back of the wall of the pharynx. The patient had had a mild degree of difficulty of breathing (probably from gathering of mucus in the throat).

The size of the tumor, its broad attachment with its, of late, rapid growth, spoke plainly for its malignant character. Carcinoma appears very seldom primarily in this part of the tongue ; the soft character of the tumor together with the absence of ulceration and absence of infiltration of the surroundings, spoke also against cancer. There was, therefore, greater reason to suspect that here was a case of sarcoma. One disease which in such a case might lead to a faulty diagnosis is lues—namely, with an unbroken gumma. No other symptoms pointing to this disease were found, however, and as well as could be ascertained, none had ever appeared. The iodide of potassium treatment, which, however, was continued a short time, gave a negative result.

Meantime the diagnosis was soon settled by microscopical examination, the only way in such a case a certain diagnosis can be determined.

The excised piece was examined at the Pathological Investigation Bureau (by Dr. Vestbery), and the swelling ascertained to be a sarcoma.

When thus the microscopical investigation had exhibited the malignant character of the growth, there was, in case a bloody operation was determined on, nothing less severe to propose to the patient than the removal of the entire tongue clear up to the epiglottis. The position of the tumor rendered it impossible in any other way completely to remove the new growth and thus prevent its reappearance. But even after such an operation no one can with certainty predict that the disease may not return. The attempt must be regarded as dangerous to life, and lastly, the patient's condition after such an operation particularly grievous, especially for a patient who, like this one, has not been accustomed to deprivations and sufferings.

I would not even propose this operation to the patient, but spoke only of it to her husband. When he heard of the dangers of the operation, what was to be won by it, and what the condition of the patient in the most favorable event would be after it, he would not consent that it should be proposed to his wife.

When thus the case might be considered an inoperable one, I proposed as the only thing which might be tried with any promise of success the injections of pyoktanin. Before proceeding further I advised the patient and her husband to obtain the opinion of Dr. Berg also. The result of his examination in company with Dr. Hakanson and myself was that he also recommended the pyoktanin treatment.

A preparatory operation of tracheotomy was made the 28th of January at the Sofia Home, as there was reason to fear that the tumor and its surroundings would swell so much after each injection that it would cause hindrance to respiration, or that possibly an edema of the glottis might make its appearance.

The 2d of February the injections of pyoktanin commenced and were continued every other or every third day ; 1 to 2 grammes of the solution (1.500) was injected each time. The injections were made by the guidance of a mirror and with long and crooked syringe points, manufactured for the special purpose, and which were inserted about 1 c. m. from the attachment of the growth and carried obliquely into the tumor.

When the injections did not cause any considerable swelling of the root of the tongue or any glottis edema, the canula was removed a week and a half afterward.

On the 15th of February, after five injections, the tumor was seen to be diminished and as if more plainly peduncleated. By digital examination its attachment was felt to be incomparably smaller than at the first examinations. While at that time the tumor seemed to be elevated, with overreaching edges only in certain places of an attachment nearly as broad as the circumference of the tumor, it is now felt to be plainly peduncleated, like a mushroom, which can be bent over to all sides upon a small stem.

On the 18th of February, after six injections, Dr. Hakanson made an excision of the elevated part of the tumor with a galvano-caustic snare. The tumor was the size of half a walnut, the stem scarcely 1 c. m. in diameter. The tumor divided into three lobes; in its frontal part, yellow ; gray on the outside ; at the back more reddish, as if transparent. Even now a pathological microscopic investigation was made at the Investigation Bureau, which justified the diagnosis.

On March 18th, the second day after the foregoing excision, a smaller mass, about the size of a coffee bean, had shot up from the left part of the tumor. This has gradually increased till it is now half as large as the one of the 18th of February. The patient has now had in all eighteen injections. Excision with galvano-caustic snare. The transverse section of the peduncle was smaller than that of the 18th of February. The following day, the 19th, the attachment of the stem was cauterized with a galvano-caustic burner. During the two following days the patient suffered slight pain in the left side of the tongue and the throat. Afterward the injections of pyoktanin were continued, of which thirty-one altogether were given, till the 26th of April, 1893, during the last part of the time with five or six days' interval. After the 18th of March there was no appearance of any further growth of the tumor, but the base of the tongue seemed for a while to be swollen.

I examined the patient last on the 6th of February, 1894, and there was no appearance of relapse. She has been well the whole time, without any feeling from her former trouble. She finds herself continuing well, without sign of the return of the disease, at the end of April, when this was printed.

